# Identification and validation of Golgi apparatus-related signature for predicting prognosis and immunotherapy response in breast cancer

**DOI:** 10.1007/s00432-024-05612-w

**Published:** 2024-02-01

**Authors:** Xin Chen, Pengting Tang, Ying Kong, Deqin Chen, Kejun Tang

**Affiliations:** 1 Department of Surgery, Women’s Hospital School of Medicine Zhejiang University, Hangzhou, 310003 Zhejiang China; 2 Department of Surgery, Ninghai Maternal and Child Health Hospital, Ninghai, 315600 Zhejiang China

**Keywords:** Golgi apparatus, Breast cancer, Immunotherapy, Prognosis, Nomogram

## Abstract

**Background:**

The Golgi apparatus plays a pivotal role in various aspects of cancer. This study aims to investigate the predictive value of Golgi apparatus-related genes (GARGs) in breast cancer prognosis and immunotherapy response evaluation.

**Methods:**

Transcriptional and clinical data from the TCGA-BRCA cohort and GSE96058 cohort were utilized to construct and validate a prognostic model for breast cancer using Cox regression analysis. Differences in immune landscape, somatic mutations, gene expression, drug sensitivity, and immunotherapy response between different risk groups were assessed. A prognostic nomogram for breast cancer was further developed and evaluated. qPCR and single-cell sequencing analyses were performed to validate the expression of GARGs.

**Results:**

A total of 394 GARGs significantly associated with breast cancer prognosis were identified, leading to the construction of a prognostic risk feature comprising 10 GARGs. This feature effectively stratified breast cancer patients into high-risk and low-risk groups, with the high-risk group exhibiting significantly worse prognosis. Meanwhile, significant differences in clinicopathological features, immune infiltration, drug sensitivity, and immunotherapy response were observed between the high- and low-risk groups. The constructed nomogram incorporating these factors showed superior performance in prognostic assessment for breast cancer patients. Ultimately, the utilization of qPCR and single-cell sequencing techniques substantiated the disparate expression patterns of these prognostic genes in breast cancer.

**Conclusion:**

Our findings demonstrate that a prognostic risk feature derived from GARGs holds promising application potential for predicting prognosis and evaluating immunotherapy response in breast cancer patients.

**Supplementary Information:**

The online version contains supplementary material available at 10.1007/s00432-024-05612-w.

## Introduction

Breast cancer is a prevalent malignant neoplasm among women and a leading cause of female mortality worldwide. Recent reports indicate an annual diagnosis of approximately 2.3 million cases, with a mortality rate of around 450,000 (Ferlay et al. 2020; Sung et al. 2021). Despite significant advancements in early detection and treatment, there remains a subset of patients with poor prognosis. Prognostic factors for breast cancer include age, tumor size, lymph node metastasis, and traditional clinical characteristics (Britt et al. 2020; Khan et al. 2021; Metcalfe et al. 2010). However, these factors fail to fully explain the variations in patient outcomes. The advent of genomics and bioinformatics technologies has shed light on the association between genetic variations and breast cancer prognosis (Shiovitz and Korde 2015). These genetic variations may involve critical biological processes such as signaling pathways (Chang et al. 2020), DNA repair (Wengner et al. 2020), and cell-cycle regulation (Repo et al. 2020). By analyzing and evaluating these genes, we can gain insights into the molecular mechanisms underlying breast cancer development and progression, ultimately guiding personalized treatment approaches.

The Golgi apparatus is a vital cellular organelle that plays pivotal roles in cellular functions and regulation (Liu et al. 2021). Recent studies have revealed a close connection between the Golgi apparatus and various diseases, including breast cancer (Luchsinger et al. 2018). As a central hub for intracellular substance transport, synthesis, and modification, the Golgi apparatus participates in crucial biological processes such as protein synthesis, modification, and localization (Kulkarni-Gosavi et al. 2019). Emerging evidence suggests that aberrant Golgi apparatus function is closely associated with breast cancer initiation and progression. Notably, the Golgi apparatus contributes significantly to tumor cell proliferation, metastasis, drug resistance, among other key aspects (Howley et al. 2018; Kajiho et al. 2016; McKinnon and Mellor 2017). Furthermore, certain GARGs exhibit abnormal expression or mutations in breast cancer and hold potential value in prognostic assessment (Ijuin et al. 2016). Thus, comprehending the intricate relationship between the Golgi apparatus and breast cancer holds paramount significance in unraveling its underlying mechanisms, identifying novel therapeutic targets, and enhancing patient prognostication.

In this study, we conducted an in-depth analysis of the expression and prognostic relevance of GARGs in breast cancer, utilizing publicly available databases. Furthermore, we constructed and evaluated breast cancer prognostic risk features based on these genes, elucidating their associations with clinical pathological characteristics, immune landscape, drug sensitivity, and response to immune therapy. The findings from this research provide valuable guidance for the clinical management of breast cancer.

## Materials and methods

### Data collection and preprocessing

Clinical information and mRNA expression profiles data of the TCGA-BRCA project were acquired from The Cancer Genome Atlas (TCGA, https://portal.gdc.cancer.gov/) database. Cases lacking complete clinical information or prognosis data were excluded, resulting in a final cohort of 869 breast cancer cases. For model validation purposes, the GSE96058 dataset was obtained from the Gene Expression Omnibus (GEO, https://www.ncbi.nlm.nih.gov/geo/) database. This dataset encompasses transcriptome and corresponding prognosis data for 3409 breast cancer cases. GARGs were retrieved from the GOCC_GOLGI_APPARATUS gene set within the MSigDB (https://www.gsea-msigdb.org/) database, which comprises a collection of 1643 GARGs. Single-cell RNA seq data were obtained by TISCH (http://tisch.comp-genomics.org/).

### Risk signature construction and evaluation

We initially assessed the significant association between GARG and breast cancer prognosis using univariate Cox regression analysis. Genes with a *p* value < 0.05 were selected for subsequent lasso Cox regression analysis to mitigate the risk of overfitting. This analytical approach was implemented employing the glmnet R package (Tay et al. 2023). Subsequently, independent prognostic GARGs were identified through multivariate Cox regression analysis, and a multi-gene risk feature was formulated utilizing the following formula: riskscore = $\sum {(g_i \times {\text{coef}}i{)}}$, where *i* denotes the number of genes, *g*_*i*_ represents the expression level of the *i*th gene, and coef_*i*_ represents the coefficient associated with the *i*th gene. The cohort was dichotomized into high-risk and low-risk groups based on median values for survival analysis, and a gene chromosome localization plot was generated employing the RCircos R package (Zhang et al. 2013). To assess the prognostic predictive performance of the risk feature, receiver operating characteristic curves (ROC curves) were employed.

### Immune landscape analysis

The CIBERSORT R package was employed to quantify the infiltration levels of 22 distinct immune cell types within the tumor tissue (Newman et al. 2019). Subsequently, a comparative analysis was conducted to assess the disparities in immune infiltration between the high-risk and low-risk groups. Furthermore, an investigation into the correlation between risk genes and tumor immune cell infiltration was performed.

### Gene set enrichment analysis

Gene set enrichment analysis was conducted using the clusterProfiler R package (Wu et al. 2021), focusing on the enrichment of biological processes derived from Gene Ontology and pathways sourced from KEGG. To account for multiple testing, the Benjamini–Hochberg correction method was employed, with a significance threshold set at *p* < 0.05.

### Mutation analysis

The somatic mutation data of the TCGA-BRCA project were retrieved from the TCGA database. The maftools package was utilized for comprehensive mutation analysis and visualization purposes (Mayakonda et al. 2018).

### Drug sensitivity analysis

The pRRophetic R package was employed to assess the sensitivity of eight drugs, namely cisplatin, doxorubicin, metformin, methotrexate, paclitaxel, sorafenib, vinorelbine, and vorinostat (Geeleher et al. 2014). The disparities in drug sensitivity between the high-risk and low-risk groups were compared and analyzed.

### Immunotherapy response analysis

The Tumor Immune Dysfunction and Exclusion (TIDE) algorithm was employed to assess the response to immune therapy. Specifically, the normalized transcriptomic data were utilized as input for the TIDE website (http://tide.dfci.harvard.edu/) to compute TIDE scores, Cancer-Associated Fibroblasts (CAF), Dysfunction, and Exclusion scores. Subsequently, a comparative analysis of the disparities in immune therapy response between the high-risk and low-risk groups was conducted.

### Nomogram construction and evaluation

Univariate and multivariate Cox regression analyses were conducted on the risk score derived from GARG, in conjunction with other pertinent clinical pathological features. Independent prognostic factors exhibiting a significance level of *p* < 0.05 were meticulously chosen to formulate a comprehensive nomogram. Subsequently, the performance of the nomogram was meticulously assessed through the utilization of calibration curves, decision curves, and ROC curves. The construction of the nomogram was facilitated by employing the rms R package (Harell 2023), while the rmda R package was employed for conducting the decision curve analysis (Brown 201).

### Validation of the GARG-derived signature

Total RNA was extracted from breast normal or cancer cell lines MCF-7, T47D, SK-BR-3, MDA-MB-231, and BT-474 using Trizol reagent (ComWin Biotech, Beijing, China) following the manufacturer’s protocol. The extracted RNA was then reverse transcribed into cDNA using the TransScript First-Strand cDNA Synthesis SuperMix kit (TransGen Biotech, Beijing, China). Real-time quantitative polymerase chain reaction (RT-qPCR) was performed in triplicate using qPCR SYBR Green SuperMix (TransGen Biotech, Beijing, China). The relative expressions of lncRNA were normalized to β-Actin as an internal reference gene using the 2^−ΔΔCT^ method. The primer sequences used in this study can be found in supplementary Table S1.

### Statistical analysis

Data analysis was performed using the R 4.2.2 software package (Team RC 2014). The Wilcoxon test was utilized to analyze the differences between the two groups. Survival analysis was conducted by generating Kaplan–Meier survival curves and applying the log-rank test. Statistical significance for differences was defined as *p* < 0.05, indicating a significant result.

## Results

### Risk signature of breast cancer derived from GARG

**Fig. 1:**
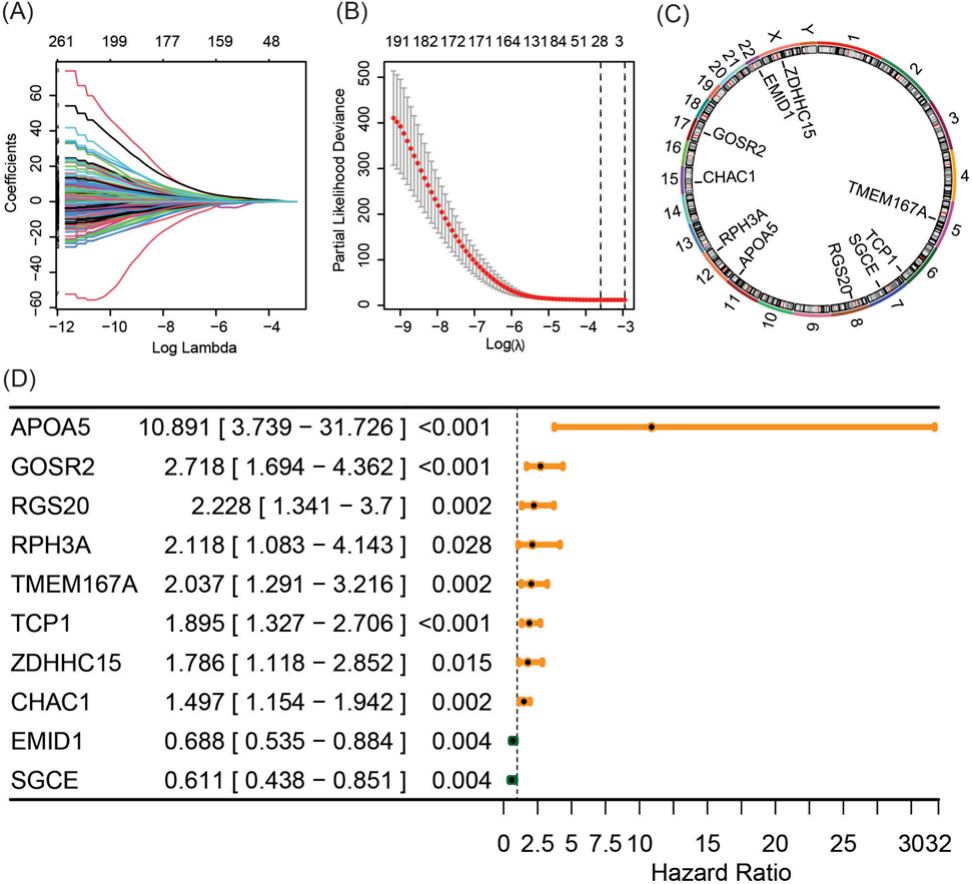
Construction of breast cancer prognosis risk features based on GARG. **A**, **B** Lasso Cox regression analysis to identify GARG associated with breast cancer prognosis. **C** Chromosomal localization of prognostic GARG identified by multivariate Cox regression analysis. **D** Forest plot of independent prognostic GARG obtained from multivariate Cox regression analysis

A total of 1643 GARG were identified, with 394 showing prognostic significance in breast cancer (Supplementary Table S2). Lasso Cox regression analysis was subsequently employed to refine the selection, resulting in the identification of 29 GARGs with prognostic value (Fig. 1A, B). Subsequent multivariate Cox analysis further narrowed down the list to ten independent prognostic GARGs (Fig. 1C), which were then utilized to construct a risk model for breast cancer prognosis. The hazard ratios of these genes are depicted in Fig. 1D, where apolipoprotein A5 (APOA5), Golgi SNAP receptor complex member 2 (GOSR2), regulator of G protein signaling 20 (RGS20), rabphilin 3A (RPH3A), transmembrane protein 167A (TMEM167A), t-complex 1 (TCP1), zinc finger DHHC-type palmitoyltransferase 15 (ZDHHC15), and ChaC glutathione specific gamma-glutamylcyclotransferase 1 (CHAC1) exhibited hazard ratios greater than 1, indicating an association with poorer survival outcomes in breast cancer patients with higher expression levels. Conversely, EMI domain containing 1 (EMID1) and sarcoglycan epsilon (SGCE) displayed hazard ratios less than 1, suggesting a favorable prognosis for breast cancer patients with higher expression levels. The risk assessment formula based on these genes is as follows: riskscore = 2.3879212 × APOA5 + 0.4032473 × CHAC1 − 0.3739642 × EMID1 + 0.9998474 × GOSR2 + 0.8010088 × RGS20 + 0.7505228 × RPH3A − 0.4931555 × SGCE + 0.6393750 × TCP1 + 0.7116043 × TMEM167A + 0.5799124 × ZDHHC15.

### Evaluation of GARG-derived risk signature

**Fig. 2:**
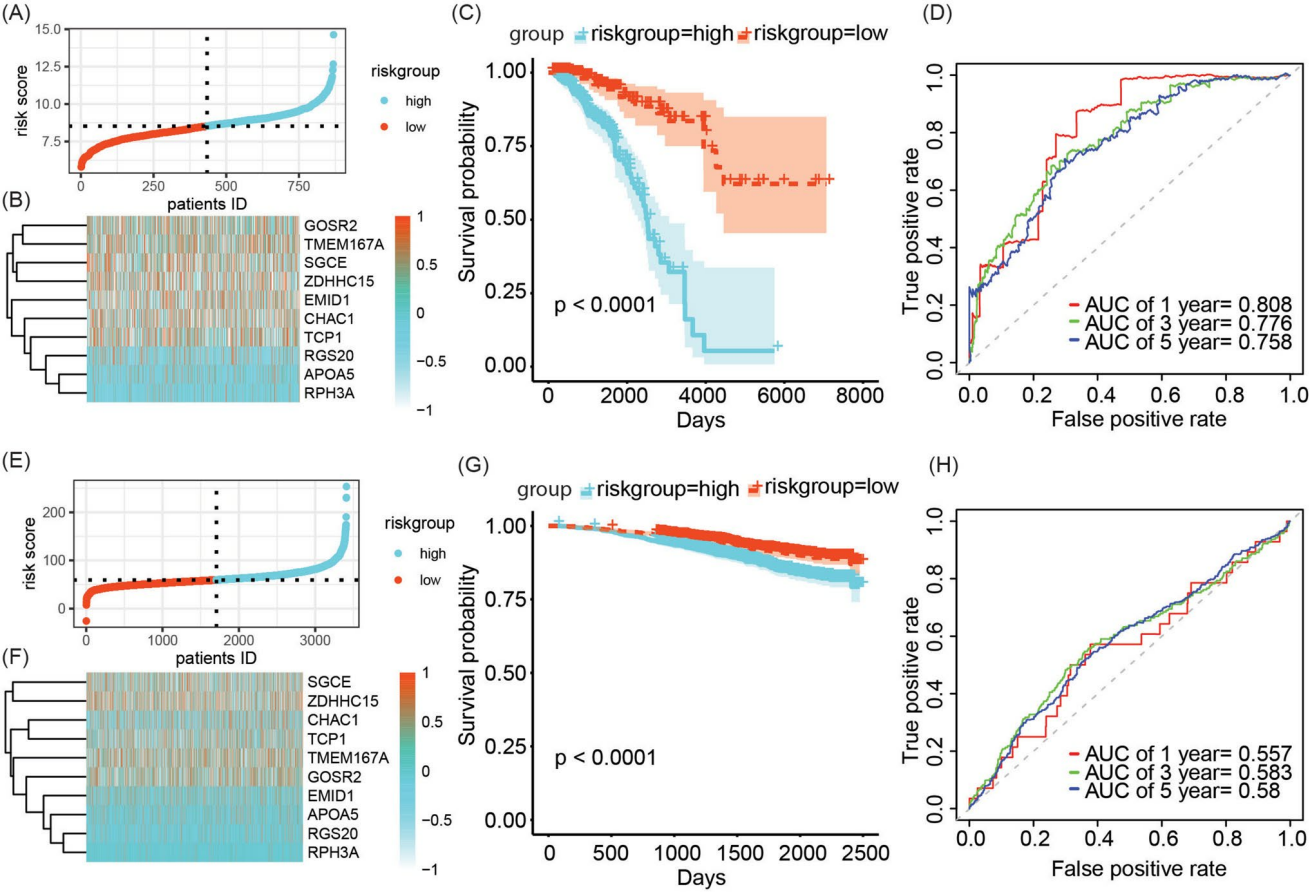
Evaluation of breast cancer risk model derived from GARG. **A**, **E** Risk group stratification based on median values in TCGA-BRCA and GSE96058 cohorts. **B**, **F** Expression heatmap of risk genes in TCGA-BRCA and GSE96058 cohorts. **C**, **G** Survival analysis of high-risk and low-risk groups in TCGA-BRCA and GSE96058 cohorts. **D**, **H** Receiver operating characteristic curves and AUC values for predicting 1-, 3-, and 5-year overall survival using risk scores in TCGA-BRCA and GSE96058 cohorts

Based on the median, the TCGA-BRCA and GSE96058 cohorts were stratified into high-risk and low-risk groups (Fig. 2A, E). The gene expression patterns in the risk features are depicted in heatmaps shown in Fig. 2B, F. Survival analysis demonstrated that patients with breast cancer in the high-risk group exhibited significantly worse prognosis compared to those in the low-risk group within the TCGA-BRCA cohort (Fig. 2C, *p* < 0.0001). Receiver operating characteristic curves were generated to assess the predictive performance of the risk score for overall survival at 1, 3, and 5 years, yielding area under the curve (AUC) values of 0.808, 0.776, and 0.756, respectively (Fig. 2D). Similarly, within the GSE96058 cohort, patients in the low-risk group displayed significantly better prognosis than those in the high-risk group (Fig. 2G, *p* < 0.0001). The AUC values for predicting overall survival at 1, 3, and 5 years based on the risk score were calculated as 0.557, 0.583, and 0.580, respectively (Fig. 2H).

### Relationship between GARG-derived risk signature and immune landscape

**Fig. 3:**
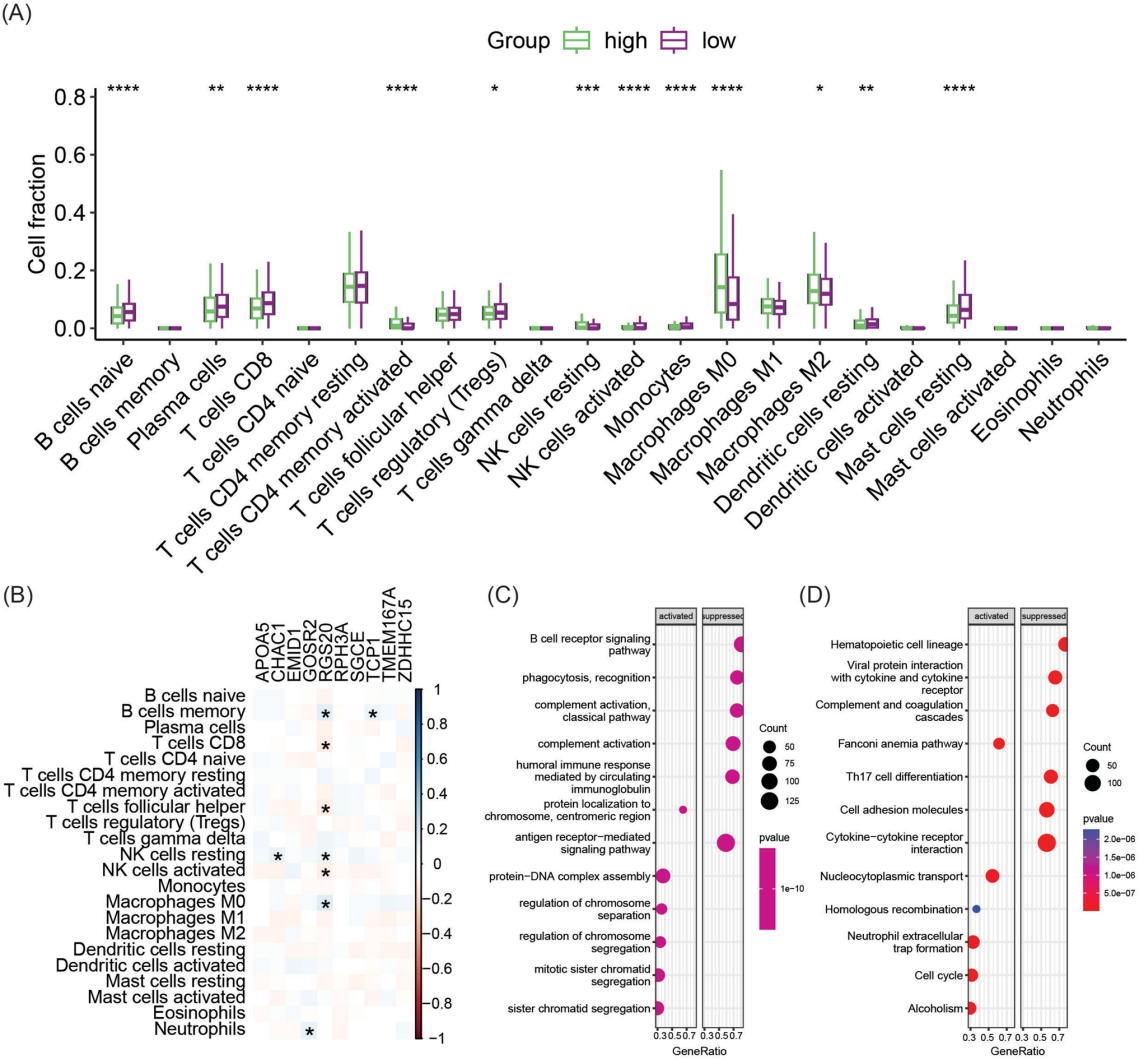
Immune landscape and gene set enrichment analysis. **A** Differential infiltration of 22 tumor immune cells between high-risk and low-risk groups. **B** Correlation between genes in risk features and immune cell infiltration. **C** Significantly suppressed and activated biological processes in high-risk group compared to low-risk group. **D** Significantly suppressed and activated KEGG pathways in high-risk group compared to low-risk group. *p < 0.05, **p < 0.01, ***p < 0.001, ****p < 0.0001

To investigate the association between GARG-derived risk features and the immune landscape, we conducted an analysis of tumor immune cell infiltration in the TCGA-BRCA cohort, comparing the differences between the high-risk and low-risk groups. Our findings revealed that patients in the low-risk group exhibited higher levels of naive B cells, plasma cells, CD8 T cells, resting dendritic cells, and resting mast cells compared to those in the high-risk group. Conversely, lower levels of M0 and M2 macrophage infiltration were observed in the low-risk group (Fig. 3A). Furthermore, correlation analysis demonstrated significant associations between gene expressions of CHAC1, GOSR2, RGS20, and TCP1 with immune cell infiltration. Notably, RGS20 exhibited associations with multiple immune cell infiltrations (Fig. 3B).

Gene set enrichment analysis further elucidated that immune-related biological processes were significantly suppressed in high-risk patients compared to their low-risk counterparts. Specifically, processes such as antigen receptor-mediated signaling pathway and humoral immune response mediated by circulating immunoglobulin were notably suppressed (Fig. 3C). Moreover, pathways related to Th17 cell differentiation and cytokine–cytokine receptor interaction were inhibited in these patients. Conversely, pathways associated with cell-cycle regulation and neutrophil extracellular trap formation were significantly activated (Fig. 3D).

### Treatment response and somatic mutation features in high-risk and low-risk groups

**Fig. 4:**
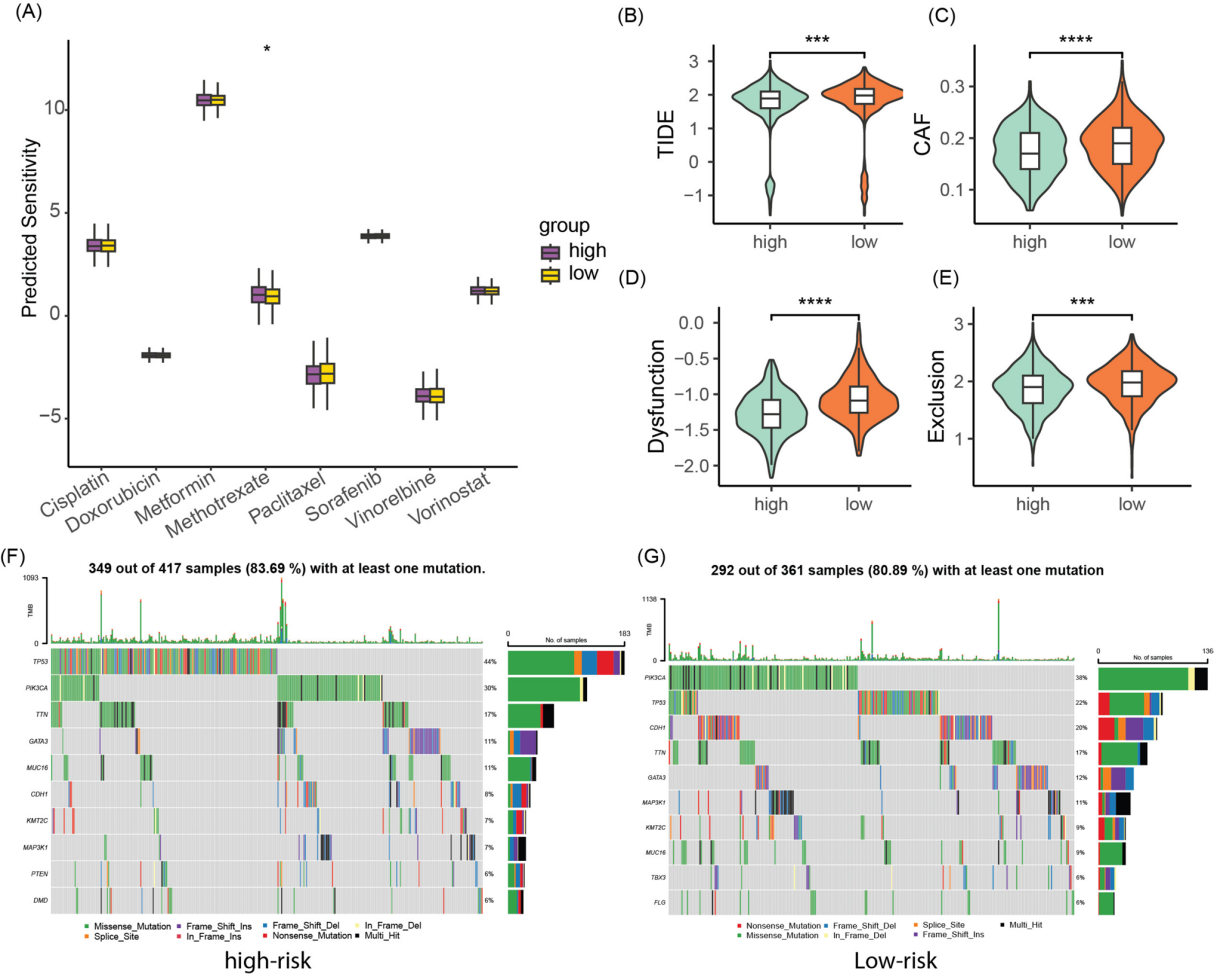
Differences in treatment response and somatic mutation features between high-risk and low-risk groups. **A** Sensitivity differences to eight chemotherapy drugs between high-risk and low-risk groups. **B**–**E** Differences in TIDE, CAF, Dysfunction, and Exclusion scores between high-risk and low-risk groups. **F**, **G** Oncoplots of somatic mutations in patients from high-risk and low-risk groups. *p < 0.05, ***p < 0.001, ****p < 0.0001

Drug sensitivity analysis revealed distinct variations in the response to methotrexate between high-risk and low-risk patients among the eight drugs investigated. Notably, low-risk patients exhibited higher sensitivity to methotrexate, indicating a greater likelihood of favorable therapeutic outcomes upon methotrexate treatment (Fig. 4A). To assess the potential efficacy of immunotherapy, we employed TIDE software to evaluate the response of high-risk and low-risk populations. Elevated TIDE prediction scores are indicative of increased immune evasion, suggesting reduced responsiveness to immunotherapeutic interventions. Strikingly, low-risk patients displayed higher TIDE scores for CAF, dysfunction, and exclusion compared to their high-risk counterparts (Fig. 4B–E). These findings suggest that high-risk patients may derive greater benefits from immunotherapy. To compare mutational genes, we listed the top ten mutational genes in both risk groups, respectively. We found that TP53, PIK3CA, TTN, GATA3, MUC16, CDH1, KMT2C, MAP3KA, PTEN, and DMD were the top ten frequent mutational genes in the high-risk group (Fig. 4F), while PIK3CA, TP53, CDH1, TTN, GATA3, MAP3K1, KMT2C, MUC16, TBX3, and FLG belonged to the top ten frequent mutational genes in the low-risk group (Fig. 4G). Furthermore, substantial differences in the patterns and frequencies of mutations among these genes were discerned between the two study groups, attesting to a potentially distinct molecular profile.

### The relationship between GARG-derived risk score and clinicopathological features

**Fig. 5:**
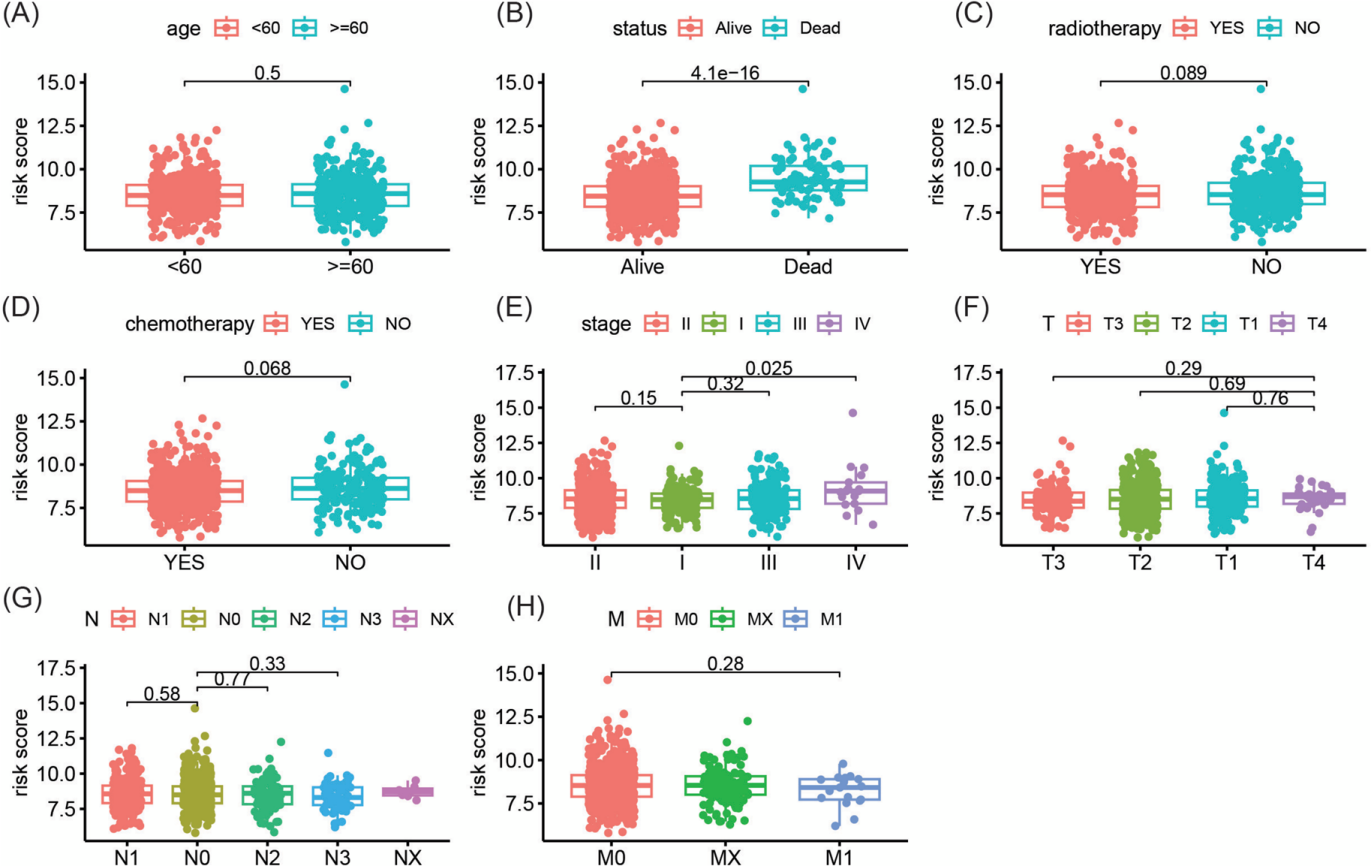
Relationship between risk score derived from GARG and clinical pathological features. **A**–**H** Differences in risk scores among different age groups, survival outcomes, radiotherapy/chemotherapy status, clinical stage, and TNM stage

We conducted a comprehensive analysis to evaluate the variations in risk scores across distinct clinicopathological subgroups (Fig. 5A–H). Notably, our findings revealed a significant disparity in risk scores between deceased and surviving patients, with the former exhibiting markedly higher risk scores (*p* = 4.1e−16). Furthermore, patients diagnosed with stage IV exhibited significantly elevated risk scores compared to those diagnosed with stage I. However, no other notable discrepancies in risk scores were observed among the remaining clinicopathological subgroups.

### Nomogram for breast cancer prognostic evaluation based on GARG-derived risk score

**Fig. 6:**
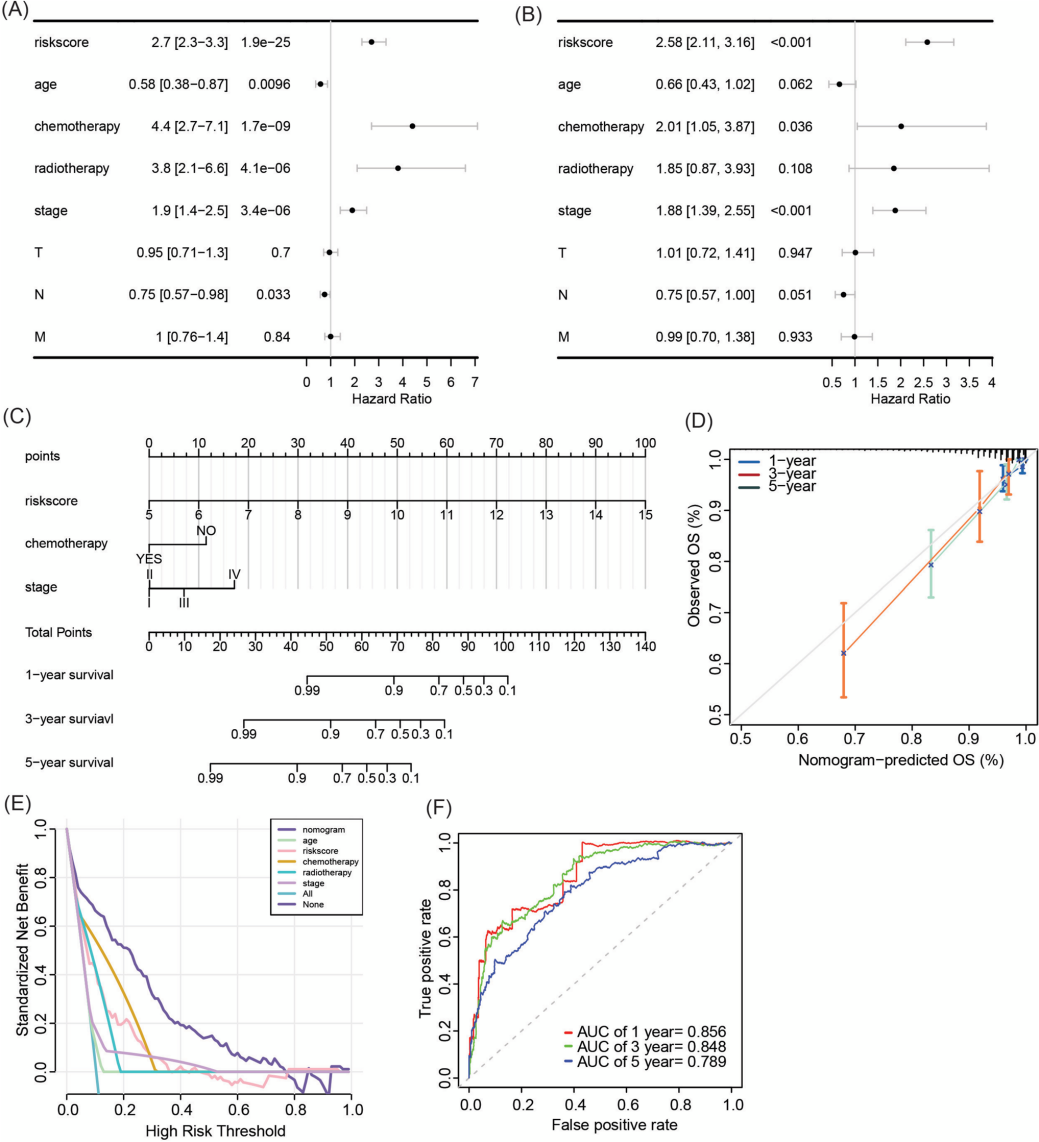
Construction and evaluation of breast cancer prognosis nomogram. **A**, **B** Univariate and multivariate Cox regression analysis of risk score and clinical pathological features. **C** Nomogram composed of risk score, chemotherapy, and clinical stage to predict 1-, 3-, and 5-year overall survival in breast cancer patients. **D**–**F** Calibration curve, decision curve, and receiver operating characteristic curve for evaluating the prognostic performance of the nomogram

Univariate Cox regression analysis was performed to identify prognostic factors for breast cancer patients, including the GARG-derived risk score, age, chemotherapy/radiotherapy, clinical stage, and N stage (Fig. 6A). Among these variables, the risk score, chemotherapy/radiotherapy, and clinical stage were found to be independent prognostic factors for breast cancer (Fig. 6B). Subsequently, a nomogram model incorporating these three independent prognostic factors was constructed for breast cancer prognostic evaluation (Fig. 6C). The calibration curve (Fig. 6D) demonstrated excellent concordance between predicted and observed values of overall survival at 1, 3, and 5 years in the TCGA-BRCA cohort. Notably, compared to individual prognostic factors, the nomogram exhibited a higher net benefit in predicting overall survival at 1 year in the TCGA-BRCA cohort, indicating its superior performance over individual prognostic factors (Fig. 6E). Receiver operating characteristic analysis indicated that the area under the curve values for predicting overall survival at 1, 3, and 5 years in the TCGA-BRCA cohort were, respectively, determined as 0.856, 0.848, and 0.789 (Fig. 6F).

### Validation of GARG expression in risk signature

**Fig. 7:**
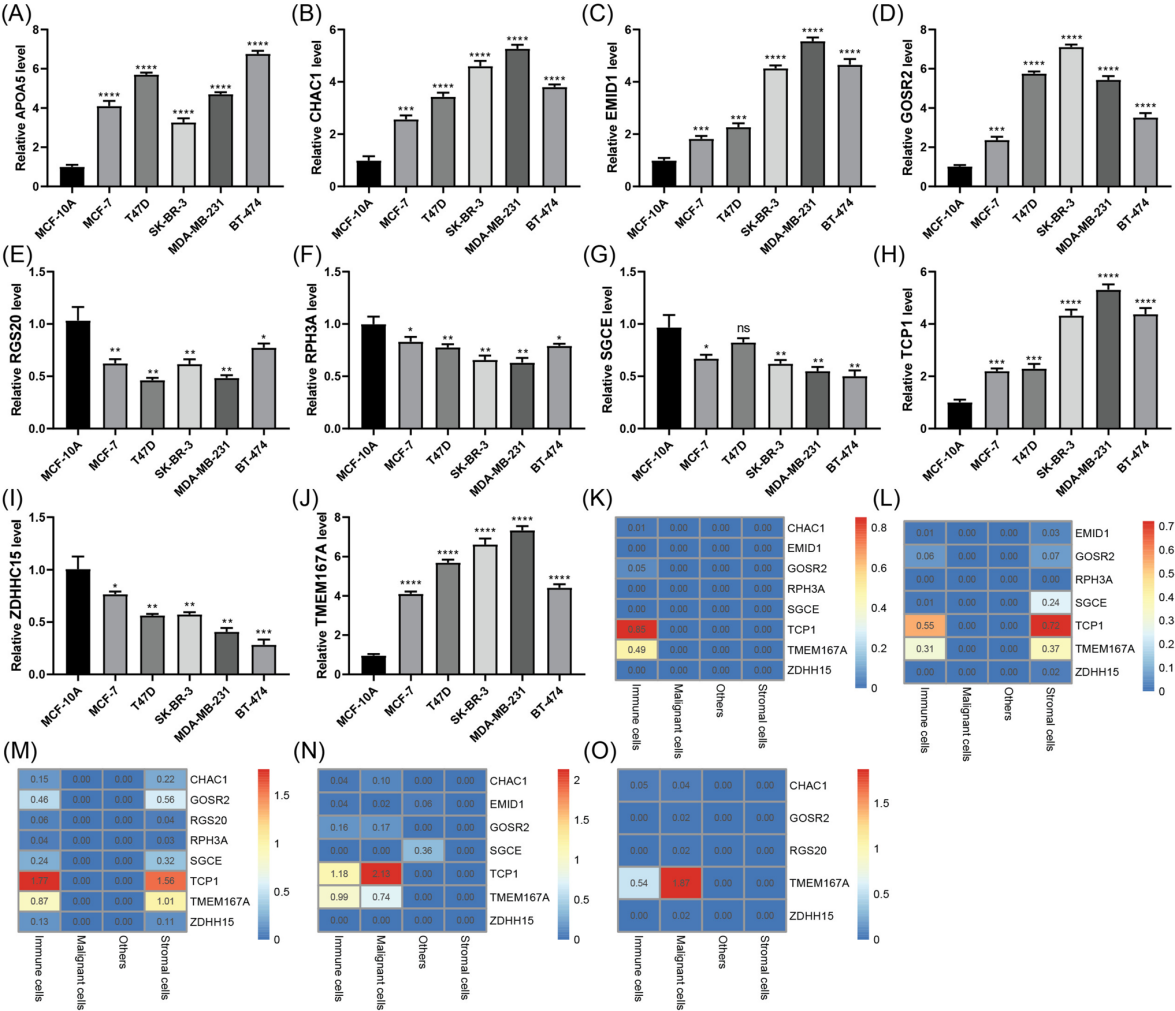
Validation of GARG expression in risk features. **A**–**J** Comparative analysis of GARG expression in breast cancer cell lines and normal controls. **K**–**O** Heatmaps depicting the expression pattern of GARG across different cell types in the TISCH database datasets, including GSE114727_10X, GSE114727_inDrop, GSE138536, GSE143423, and SRP114962

The expression levels of GARG were meticulously validated in both normal and cancerous breast cells using the highly sensitive RT-qPCR technique. Comparative analysis with the well-established MCF-10A reference cell line (Fig. 7A–J) revealed a significant upregulation of APOA5, CHAC1, EMID1, GOSR2, TCP1, and TMEM167A in breast cancer cell lines. Conversely, RGS20, RPH3A, SGCE, and ZDHHC15 exhibited a substantial downregulation in their expression levels within the corresponding cancer cell lines. Moreover, employing cutting-edge single-cell sequencing analysis provided further insights into the expression patterns of GARG within risk features. Notably, GARG was found to be expressed across tumor-infiltrating immune cells, malignant cells, and stromal cells. Of particular significance were TCP1 and TMEM167A, which displayed specific expression profiles in both immune cells and stromal cells across multiple datasets (Fig. 7K–O). These compelling findings underscore the pivotal role of GARG as a key regulatory element implicated in breast cancer pathogenesis while highlighting its potential as a promising biomarker for diagnostic purposes and therapeutic targeting.

## Discussion

The Golgi apparatus plays a critical role in cancer pathogenesis, and its functional abnormalities have been implicated in pivotal processes including cellular proliferation, metastasis, and drug resistance. Extensive research has demonstrated that molecular subtyping and prognostic risk assessment based on GARGs hold substantial promise in accurately predicting prognosis and response to immune therapy in hepatocellular carcinoma. Motivated by these findings, our study aimed to investigate the association between GARGs and breast cancer prognosis. In addition, we sought to construct a comprehensive multi-gene risk feature comprising ten GARGs. Our analyses revealed that this novel risk feature exhibited robust prognostic value and displayed significant correlations with tumor immune cell infiltration as well as response to immune therapy.

Within this risk feature, APOA5 encodes an apolipoprotein that plays a pivotal role in the regulation of plasma triglyceride levels. Notably, studies have demonstrated a higher prevalence of APOA5 copy number loss in intraductal tumors among East Asian women under the age of 50, suggesting its diverse involvement in tumor biology through immunomodulation within the tumor microenvironment (Lin et al. 2021). GOSR2 encodes a transport membrane protein responsible for mediating protein transportation between cis- and trans-Golgi compartments. However, further investigations are warranted to elucidate its precise implications in cancer. RGS20 has been extensively validated as a participant in cancer initiation and progression. For instance, it has been shown to facilitate tumor advancement in penile cancer by modulating PI3K/AKT signaling activation (Shi et al. 2022). In addition, RGS20 promotes proliferation and migration in bladder cancer through activation of the NF-κB signaling pathway (Li et al. 2019). Neutrophil polarization has been associated with lung metastasis in triple-negative breast cancer (Wang et al. 2020), while RPH3A plays a critical role in neutrophil polarization (Ren et al. 2020) and may contribute significantly to breast cancer metastasis. TMEM167A, located within the Golgi apparatus, regulates vesicular transport to control growth factor signaling activity and determines invasiveness of wild-type p53 glioblastoma (Segura-Collar et al. 2020). TCP1 encodes a molecular chaperone protein that modulates the PI3K/AKT/mTOR signaling pathway, thereby promoting ovarian cancer cell proliferation (Weng et al. 2021) and enhancing drug resistance in acute myeloid leukemia (Chen et al. 2021). ZDHHC15 has been identified as a promoter of glioblastoma malignancy and can serve as a novel prognostic biomarker for glioblastoma patients (Liu et al. 2023). CHAC1 is associated with ferroptosis and serves as a prognostic factor across various cancers, including renal clear cell carcinoma (Li et al. 2021) and stomach adenocarcinoma (Xiao et al. 2022). EMID1 represents a potential candidate gene that promotes metastasis and exhibits upregulation in lung adenocarcinoma, correlating with improved prognosis and immune infiltration (Shao et al. 2022). Lastly, SGCE stabilizes EGFR to promote breast cancer stem cells, offering new insights into overcoming the challenges associated with targeting EGFR in current clinical trials (Zhao et al. 2020).

The dysregulation of Golgi dynamics has been demonstrated to profoundly impact the tumor microenvironment and immune landscape, thereby potentiating the invasive and metastatic capacities of cancer cells. In this study, we meticulously examined the disparities in immune infiltration patterns between high-risk and low-risk patient cohorts, while concurrently elucidating the interplay between risk features derived from GARG and the intricate immune landscape. Our correlation analysis unveiled numerous significant associations linking the expression levels of RGS20 with distinct immune cell infiltrates, thereby implying its potential regulatory role within the tumor immune microenvironment. The PI3K/AKT signaling pathway, a pivotal cellular signaling cascade governing diverse biological processes such as cell survival, proliferation, differentiation, and metabolic regulation (Porta et al. 2014), may potentially underlie the observed connections between RGS20, TCP1, and immune cell infiltration (Shi et al. 2022; Weng et al. 2021; Chen et al. 2021). Consequently, to comprehensively investigate both the expression patterns and immunoregulatory functions of these genes within tumor tissues, future research endeavors should consider integrating single-cell sequencing methodologies with rigorous biological experimentation.

Breast cancer immunotherapy represents a promising therapeutic avenue, seeking to harness the patients’ immune system to combat breast cancer. Notably, tumor-associated antigen vaccines, CAR-T cell therapy, and immune checkpoint inhibitors have emerged as pivotal modalities within the realm of breast cancer immunotherapy (Gaynor et al. 2022; Huang et al. 2022; Xu et al. 2021). Nevertheless, the heterogeneity in treatment response among patients necessitates a deeper understanding of factors influencing therapeutic outcomes. In this study, we have identified that risk features derived from GARG hold potential as predictive indicators for immunotherapeutic response in breast cancer patients. This finding bears significant clinical implications by enabling the provision of more effective and personalized treatment strategies for individuals afflicted with breast cancer.

Finally, risk features derived from GARG were identified as significant independent prognostic factors for breast cancer, in addition to chemotherapy and clinical staging. These features were utilized to develop a prognostic assessment nomogram model that exhibited superior predictive accuracy compared to other established independent prognostic factors. However, it is important to acknowledge the limitations of this study. Despite validating the constructed model using a GEO dataset, its performance fell short of optimal standards, highlighting the necessity for further validation employing additional clinical samples. Moreover, comprehensive exploration of the biological functions of this prognostic GARG is warranted, particularly with regard to its role within the tumor immune microenvironment.

## Conclusion

In conclusion, this study has successfully elucidated the prognostic significance of GARG in breast cancer. We have meticulously constructed and assessed risk features derived from GARG, establishing their associations with various clinical pathological characteristics, immune landscape, drug sensitivity, and immune therapy response. Moreover, we have developed a robust breast cancer prognostic assessment nomogram by integrating these features with other established independent prognostic factors. The findings of this study lay a solid groundwork for future advancements in personalized treatment strategies for breast cancer.

### Supplementary Information

Below is the link to the electronic supplementary material.

Supplementary file1 (XLSX 42 KB)

## Data Availability

The datasets generated during and/or analyzed during the current study are available from the corresponding author on reasonable request.

## Abbreviations

GARGs: Golgi apparatus-related genes
TCGA: The Cancer Genome Atlas
GEO: Gene expression omnibus
ROC: Receiver operating characteristic
TIDE: Tumor immune dysfunction and exclusion
CAF: Cancer-associated fibroblasts
APOA5: Apolipoprotein A5
GOSR2: Golgi SNAP receptor complex member 2
RGS20: Regulator of G protein signaling 20
RPH3A: Rabphilin 3A
TMEM167A: Transmembrane protein 167A
TCP1: T-complex 1
ZDHHC15: Zinc finger DHHC-type palmitoyltransferase 15
CHAC1: ChaC glutathione specific gamma-glutamylcyclotransferase 1
EMID1: EMI domain containing 1
SGCE: Sarcoglycan epsilon
AUC: Area under the curve
TP53: Tumor protein p53
PIK3CA: Phosphatidylinositol-4,5-bisphosphate 3-kinase catalytic subunit alpha
